# Estimating Cervical Vertebral Maturation with a Lateral Cephalogram Using the Convolutional Neural Network

**DOI:** 10.3390/jcm10225400

**Published:** 2021-11-19

**Authors:** Eun-Gyeong Kim, Il-Seok Oh, Jeong-Eun So, Junhyeok Kang, Van Nhat Thang Le, Min-Kyung Tak, Dae-Woo Lee

**Affiliations:** 1 Division of Computer Science and Engineering, Jeonbuk National University, Jeonju 54907, Korea; egg13605@gmail.com (E.-G.K.); isoh@jbnu.ac.kr (I.-S.O.); jeongeunso@jbnu.ac.kr (J.-E.S.); k1101jh@naver.com (J.K.); 2 Department of Pediatric Dentistry, Research Institute of Clinical Medicine, Jeonbuk National University, Jeonju 54907, Korea; lvnthang@hueuni.edu.vn (V.N.T.L.); aubade0823@naver.com (M.-K.T.); 3 Biomedical Research Institute, Jeonbuk National University Hospital, Jeonju 54907, Korea; 4 Faculty of Odonto-Stomatology, Hue University of Medicine and Pharmacy, Hue University, Hue 49120, Vietnam

**Keywords:** bone maturation, cervical vertebrae maturation, deep learning, lateral cephalogram

## Abstract

Recently, the estimation of bone maturation using deep learning has been actively conducted. However, many studies have considered hand–wrist radiographs, while a few studies have focused on estimating cervical vertebral maturation (CVM) using lateral cephalograms. This study proposes the use of deep learning models for estimating CVM from lateral cephalograms. As the second, third, and fourth cervical vertebral regions (denoted as C2, C3, and C4, respectively) are considerably smaller than the whole image, we propose a stepwise segmentation-based model that focuses on the C2–C4 regions. We propose three convolutional neural network-based classification models: a one-step model with only CVM classification, a two-step model with region of interest (ROI) detection and CVM classification, and a three-step model with ROI detection, cervical segmentation, and CVM classification. Our dataset contains 600 lateral cephalogram images, comprising six classes with 100 images each. The three-step segmentation-based model produced the best accuracy (62.5%) compared to the models that were not segmentation-based.

## 1. Introduction

Bone maturation is used to diagnose metabolic disorders in pediatric endocrinology and to distinguish sexual maturation characteristics, chronological age, and dental development [1]. Determining the maturation and subsequent evaluation of growth potential during preadolescence or adolescence is important. The most standard method used to estimate bone maturity is the use of a hand–wrist radiograph. Typically, the Greulich–Pyle (GP) [2] and Tanner–Whitehouse (TW) [3] methods are used.

For orthodontic treatment, a lateral cephalogram must be obtained to diagnose skeletal deformities and malocclusion in patients. If bone maturation can be estimated by lateral cephalograms, additional exposure to hand–wrist radiographs can be avoided. Expert dentists require a long time to estimate cervical vertebral maturation (CVM) and may obtain different results each time they perform the estimation.

CVM, which was proposed by Baccetti and Franchi, estimates maturation using C2, C3, and C4. After the morphological characteristics of the cervical spine are quantitatively analyzed, maturity is estimated in six stages (cervical stage, hereinafter CS1–CS6). This method was criticized for its reproducibility, as it lacked a clear explanation; however, to compensate for this, McNamara and Franchi made a user’s guide [4] that explains the CVM method in more detail. Several studies have reported that the reproducibility of the CVM method varies across observers; therefore, it is limited in its use as a clinical guide [5,6].

Deep learning-based methods using convolutional neural networks (CNNs) have recently been proposed. Deep learning models for hand radiographs show excellent performance [7,8,9]. Research on the estimation of bone maturation using lateral cephalograms is in the beginning stage. There have been various studies on the estimation of CVM using lateral cephalograms. To analyze the correlation between the morphological transformation of the cervical spine and the mandible, Gray took a landmark on the lower curve of C2 and the outlines of C3 and C4 and analyzed the shape of the cervical spine by creating a landmark distribution model [10]. Baptista used the Naive Bayes classifier [11], and Dzemidzic classified CVM using a decision tree [12] after analyzing the rectangular characteristics and the degree of concaveness in the contours of C2, C3, and C4. Moraes approached the regression problem, which outputs bone age by learning the bottom surface of C3 using a simple multi-layer perceptron [13]. Similar to Gray’s study, Kök extracted and classified features, such as the width and length of the cervical body, by marking a landmark on the outer edge of the cervical spine [14]. Amasya also considered landmarks outside C5 and classified the features by extracting them using the ratio between the lower part of the cervical body and the left and right lengths of the body [15]. In this case, artificial neural network, k-nearest neighbor, decision tree, random forest, support vector machine, and logistic regression algorithms were used as classifiers. The studies described above used classical machine learning-based methods, which are based on a manual design of features that require expert knowledge, that is, handcrafted features. This design approach involves highly laborious work and leads to suboptimal performance.

In contrast, the deep learning model learns features that adapt to the given data. Therefore, it requires less expert knowledge than classical methods. Makaremi proposed a deep learning CNN-based method. However, the method required manual segmentation of the ROI [16]. They also proposed an experiment that extracts Sobel edge features and uses them as input. These are opposite to feature learning, which is the basic principle of deep learning. Seo et al. presented a benchmarking result using six deep learning models that are variants of ResNet, GoogLenet, and MobileNet [17]. Their system is semi-automatic since it requires manual cropping of ROI as preprocessing. The proposed method in our study suggests a deep learning-based method for automatically estimating CVM from a lateral cephalogram. This study aims to propose deep learning models for estimating CVM that work in a fully automatic manner.

## 2. Materials and Methods

### 2.1. Dataset

The lateral cephalogram dataset used in this study was provided by the Jeonbuk National University Dental Hospital. The sample images were collected from 6- to 18-year-old children and adolescents who visited the Jeonbuk National University pediatric department for orthodontic treatment between 2008 and 2018. A total of 600 images were used in this experiment. The resolution of the images ranged from 1714 × 2164 to 2200 × 2200 pixels. Two specialists, one with 14 years of experience and the other with 2 years of experience at Jeonbuk National University Dental Hospital, classified images into one to six maturity levels according to the CVM method [18]. Each clinician independently evaluated the stage of cervical vertebrae age, and inconsistent results were determined through consensus. They also segmented the C2, C3, and C4 regions using a drawing software tool. A clinician with 2 years of experience conducted the segmentation, and a doctor with 14 years of experience confirmed the segmentation results. The bounding box is a rectangle drawn 200 pixels away from the top, bottom, left, and right sides of the segmentation image.

**Figure 1:**
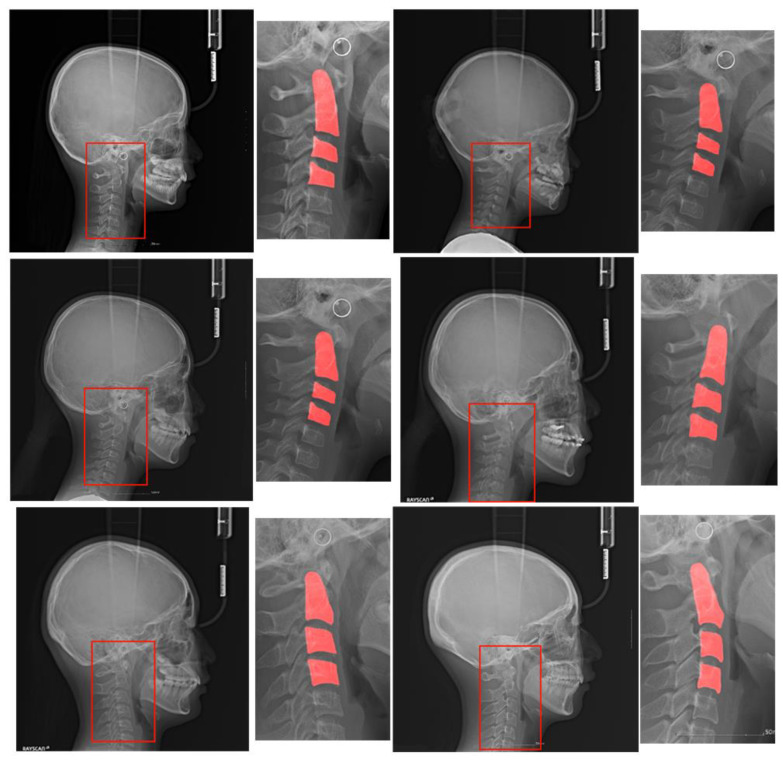
Original lateral cephalograms and regions of interest (ROIs) with segmentation regions. These pairs are examples of each of the six stages of a lateral cephalogram, and each pair has an original image and ROI with the C2, C3, and C4 segmentation regions. The red box in the original image is the ROI and it is magnified on the right side with the segmentation region. Note that cropped images have various sizes.

Figure 1 shows a pair of original images and ROIs with the C2, C3, and C4 segmentation regions for each of the six classes. The ROI was drawn as a bounding box enclosing the three regions.

The lateral cephalogram shown in Figure 1 has a width and height between 2000 pixels and 2200 pixels. The cervical spine area required in this study was approximately 100 pixels, which is very small compared to the original image. Some studies resolved this discrepancy problem by designing a suitable loss function [19,20]. We used a more direct approach that explicitly obtains C2, C3, and C4 segmentation masks. We used a binary mask with a value of 0 for the black background and 1 for the foreground.

To implement our approach, we designed three modules: ROI detector, segmentor, and classifier. These modules are specified in Section 2.1. By assembling the modules in a cascading manner, three models can be designed: model_1 (classifier model), model_2 (ROI detector–classifier model), and model_3 (ROI detector–segmentor–classifier model). These models are explained in Section 2.2.

### 2.2. Modules

#### 2.2.1. Classifier

The aim of the classifier module is to classify an input image into one of six classes according to the cervical stage. We trained the CNN to accomplish this aim. The dataset contained only 100 images per class, and since the training set was small, we used transfer learning. ResNet50 was adopted, which was pre-trained with a large natural image dataset named ImageNet [21].

During transfer learning, the fully connected layers in ResNet50 were removed, and a new fully connected layer with six output nodes whose weights were initialized randomly was attached. All weights of the convolutional layers were frozen, except those of the 2nd, 3rd, and 4th layers. The rebuilt ResNet50 was trained using the Adam optimizer and the cross-entropy loss function.

#### 2.2.2. ROI Detector

**Figure 2:**
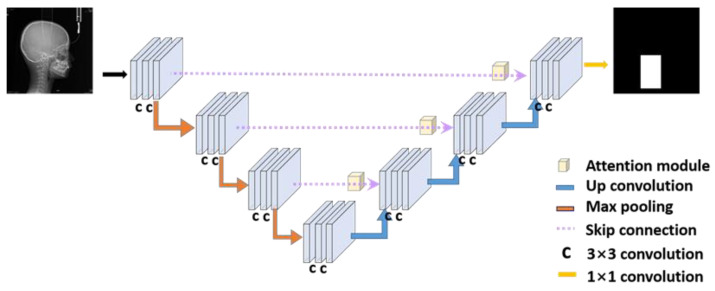
The architecture of the ROI detector module. The original X-ray image is an input in this module, and the output is an ROI binary image. The main network is attention U-Net.

As shown in Figure 1, the area occupied by the cervical vertebrae was smaller than the entire image. The problem of measuring the bone age of the cervical vertebrae depends heavily on the shapes of C2, C3, and C4 in the images. If the original image is used for classification, deep learning may classify the cervical stage based on parts other than the cervical spine, leading to poor performance. As an effective approach to remedy the problem, the ROI can be segmented around C2–C4, so that the classifier module can focus more on C2–C4. The structure of the ROI detector is shown in Figure 2.

The U-Net is a deep learning model proposed for image segmentation. It is based on fully convolutional networks, FCN, in which all layers are convolutional layers. This network was named U-Net because it was shaped like a ‘U’. It has a contracting path going down to the symmetry point and an expanding path going up from that point. In the contracting path, it captures the characteristics of the input images and reduces the size of the image. This path consists of repeated application of 3 × 3 convolutions with a rectified linear unit (ReLU), and a 2 × 2 max pooling operation for downsampling. In the expanding path, the image is expanded for accurate segmentation. There are 3 × 3 convolutions with a ReLU and upsampling of the feature map by 2 × 2 up convolution in this path. A 1 × 1 convolution is in the final layer. The border information is revised by adding the copy of the cropped feature map of the contracting path to the upsampled feature map in the expanding path [22]. This crop and copy process is called the skip connection because it brings the previous feature map.

In ROI detection, we used attention U-Net. It can be used for this module because the detection is a kind of segmentation. Attention U-Net uses an attention gate when upsampling in U-Net’s crop and copy process. It was experimentally proven to produce better results [23]. Our model in Figure 2 consists of 14 3 × 3 convolution layers, 3 2 × 2 up convolution layers, and one 1 × 1 convolution layer.

To train the attention U-net for ROI detection, a labeled dataset is essential. We used the C2–C4 segmentation dataset described in Figure 1. A rectangle is drawn at 200 pixels away from the top, bottom, left, and right sides of the C2–C4 segmented patches, and the rectangle is regarded as the ROI label. When the original radiograph is entered as an input into the attention U-net, a grayscale map is produced. Using a fixed threshold value of 0.8, the grayscale map with range [0, 1] is converted into a binary image. We used the Adam optimizer and cross-entropy loss.

#### 2.2.3. Segmentor

While the ROI detector can make a neural network focus more on the C2–C4 regions, it cannot extract the shape information of C2–C4. On a radiograph, the underside of the cervical vertebral body may appear to overlap because of the problem of the shooting angle. In this case, even if the ROI is segmented, the overlapping regions are visible. Using segmentation images represented in a binary map, a neural network can focus only on the cervical spine.

**Figure 3:**
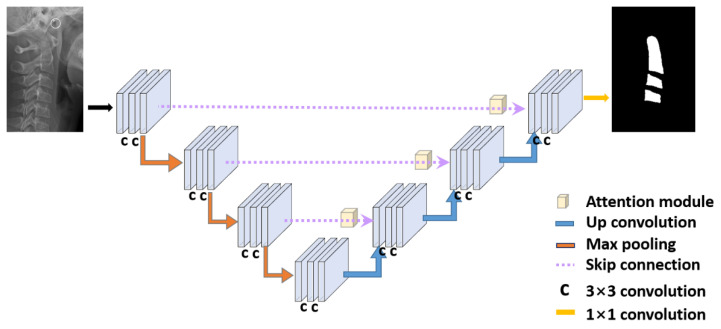
The architecture of the segmentor module. The cropped ROI image is input in this module, and the output is a cervical vertebrae segmentation image. The main network is attention U-Net.

The segmentor module receives the ROI segmented from the original image as an input and outputs the segmented image of the cervical spine as a result. When the cropped patch image is provided as an input into the network, a grayscale map is produced. Using a fixed threshold value of 0.8, the grayscale map with the range of [0, 1] is converted into a binary image. Similar to the ROI detector in the previous section, this module uses the attention U-Net, which delivers a better performance than U-Net. The structure of the segmentor shown in Figure 3 is the same as that of the ROI detector. Only the inputs and outputs were different. We used the Adam optimizer and cross-entropy loss.

### 2.3. Classification Models

**Figure 4:**
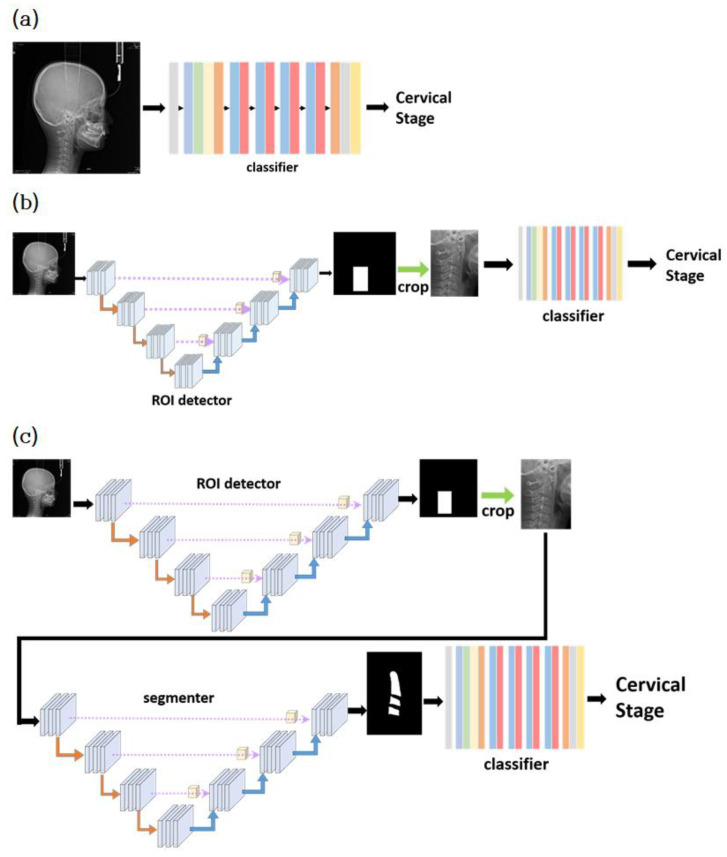
Architecture of three models: (**a**) model_1, (**b**) model_2, and (**c**) model_3.

Classification models can be made by assembling the modules in a cascading manner. The model estimates the CVM using a lateral cephalogram. The simplest model, model_1, consists of only the classifier module. The input of model_1 is the 224 × 336 image resized from the original image. The average size of the original images was approximately 2100 × 2200. The resized image fits into model_1, and model_1 predicts one of the six classes as output. Figure 4a illustrates model_1.

The second model, model_2, employs both the classifier and ROI detector modules. As shown in Figure 4b, the first part of model_2 is the ROI detector module, and the second part is the classifier module. The original image was resized to 224 × 224 and input the ROI detector. The ROI detected by the ROI detector was cropped from the original image. The cropped ROI was resized to 448 × 224 and input to the classifier module for classification.

The last model, model_3, uses all three modules described in the previous section. As shown in Figure 4c, it consists of ROI detector–segmentor–classifier modules in sequence. The ROI detector receives as input the resized image with 224 × 224 resolution. The cropped ROI was resized to 448 × 224 and input to the segmentor module. The segmentor produces the binary image, where pixels with 0 represent the background, and pixels with 1 represent the foreground (cervical vertebrae). Finally, the binary image is input into the classifier module. The classifier module converts the binary image into one of the six classes.

## 3. Results

### 3.1. Experimental Environment

The experiment was based on Python 3.6 and PyTorch 1.2.0. The experiment was performed on an Ubuntu 16.04 and NVIDIA GeForce RTX 2080 TI with 11 GB RAM. For data augmentation, we used rotation, horizontal and vertical flip, and changes in brightness, contrast, saturation, and hue. The rotation was allowed randomly between −20° and 20°. To generalize the results in this dataset, we used 5-fold cross-validation. Each fold was divided into 8:2, trained, and tested.

When assessing maturity performance, we must consider the gradual progression of body growth. Therefore, there is a correlation between the current stage and the preceding and subsequent stages. Since it is necessary to refer to the front and rear stages clinically, model accuracy is evaluated using the ε index, allowing one- or two-stage errors. ε(0) allows for no errors. ε(1) allows a one-stage error, that is, classifying into the previous or next stage is considered the correct answer. For example, when the correct maturity stage is CS3, all the model predictions of CS2, CS3, and CS4 are considered correct.

### 3.2. Evaluation of ROI Detector and Segmentor

**Figure 5:**
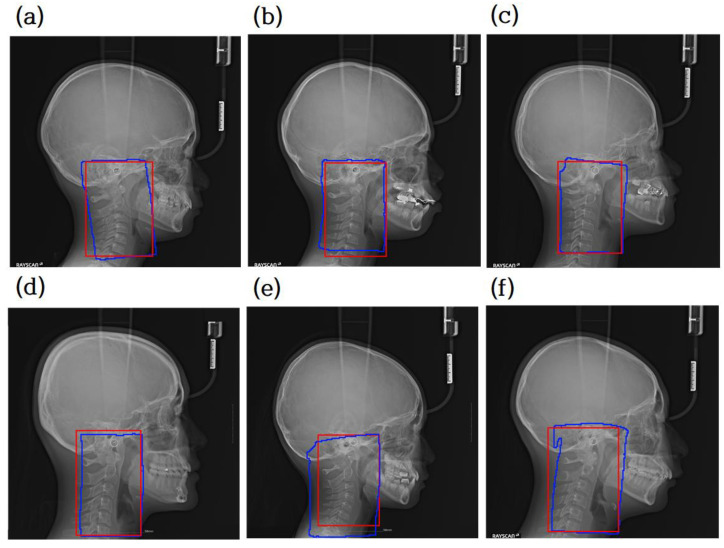
Comparison between ground truth of segmentation mask for extracting the region of interest (ROI) and prediction mask. Red is the ground truth, while blue is the predicted result. (**a**–**d**) are examples of accurate ROI detection of cervical vertebrae area. Panels (**e**,**f**) are not exactly tetragonal shaped, but they are necessary for the cervical vertebral region.

A comparison between the prediction result and ground truth is shown in Figure 5, where the red box represents the ground truth and the blue box represents the prediction result. The blue box is obtained by following the boundary of the detected region with a value of 1. Finally, the blue box is converted into a rectangle with the minimum x, y values and the maximum x, y values. The intersection over union (IOU) between the ground truth rectangle and the predicted rectangle was used to evaluate the model. IOU scores were measured and averaged over the entire test image. The average IOU score was 0.9181. As confirmed by the blue box in Figure 5, the predicted ROI includes the cervical vertebral regions C2, C3, and C4 for all the test images.

**Figure 6:**
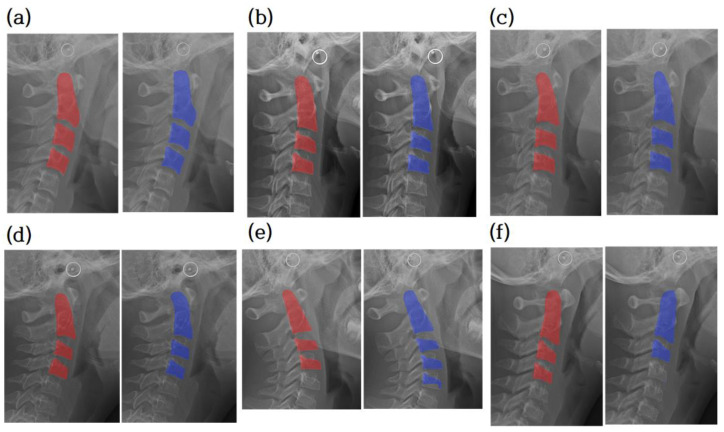
Cervical vertebrae segmentation mask. Comparison between ground truth of segmentation and prediction. The radiograph overlaid with the ground truth of the segmentation image (red). The radiograph is overlaid with the prediction (blue). (**a**–**d**) are examples of accurate segmentation of cervical vertebrae. (**e**,**f**) are examples of segmentation of less or more than three vertebrae. Note that cropped images have various sizes.

The segmentation ground truth and the output of the segmentor are compared in Figure 6. Most of the 120 test images were correctly segmented into three regions, as shown in Figure 6a–d. Nine test images were segmented with more than three regions, as shown in Figure 6e, and three test images were segmented with less than three regions, as shown in Figure 6f.

### 3.3. Evaluation of Classification Accuracy of the Three Models

Table 1 shows the classification accuracies of models 1, 2, and 3 evaluated using the ε index.

Model_3 showed the highest accuracy in terms of ε(0), which is a significant improvement over models 1 and 2. The accuracy of model_3 was 9.17% higher than that of model 1 and 3.34% better than that of model 2. In terms of ε(1), the accuracy of all three models was better by more than 30% compared to that of ε(0). The reason for the impressive improvement is that there are many cases where the stage of cervical vertebrae maturity spans between the previous and next stages of the correct answer. We must consider the gradual progression of body growth. Many cases are ambiguous as to whether a patient’s cervical vertebral maturity in the transition from CS1 to CS2 should be classified as CS1 or CS2. Therefore, ε(1) is meaningful in helping dentists’ decision making. In terms of ε(2), all the models had an accuracy of more than 98%.

**Figure 7:**
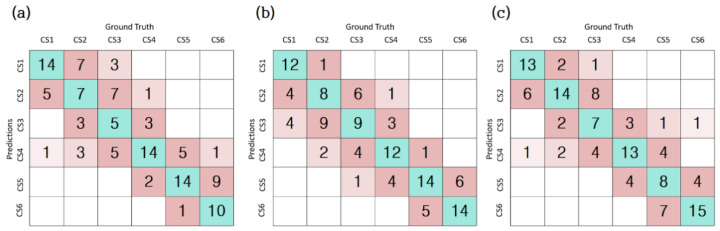
(**a**) Confusion matrix of model_1; (**b**) confusion matrix of model_2; (**c**) confusion matrix of model_3.

For the comparison of each model (Figure 7), the confusion matrix was made based on the last fold. The row of the matrix is the ground truth of the class, and the column is the class predicted by deep learning.

## 4. Discussion

Our model automatically detects the ROI and segments the C2, C3, and C4 regions, leading to a fully automatic system with a reasonable performance. The contributions of this study are as follows:

- Dataset construction: Our dataset contains 600 lateral cephalograms collected from patients visiting the Jeonbuk National University Dental Hospital, Korea. Each image is labeled with one of the six classes representing the bone maturity stage. In addition, to support segmentation-based model learning, the C2, C3, and C4 cervical vertebrae are labeled with segmentation information at the pixel level.
- The C2, C3, and C4 cervical vertebrae are very small compared to the whole image, resulting in a severe data imbalance. To solve this issue, we propose attention U-Net models that automatically detect the ROI and segment the C2, C3, and C4 regions with high accuracy.
- We propose three CNN-based classification models: a one-step model with only CVM classification, a two-step model with ROI detection and CVM classification, and a three-step model with ROI detection, cervical segmentation, and CVM classification. Their accuracies were compared with those of our dataset.

Since human maturation is continuous, the estimation of CVM is inherently a regression problem. In the medical field, the regression problem is converted into a classification problem by discretizing the continuous CVM level into six classes. Discretization incurs a barrier in obtaining a satisfactory performance even by experienced radiologists. A recent study that evaluated human performance for CVM revealed a very low reproducibility [24]. Six experts in radiology and orthodontics participated in the evaluation. The intra-rater agreement ranged from 77.0% to 87.3% and inter-rater agreement was only 42.82%. A deep learning model has no choice but to use the class labels made by human experts who are doomed to low reproducibility. It seems that there is an upper bound achievable by computer algorithms and the upper bound is far from being satisfactory. However, it does not mean the model is useless. As other medical artificial intelligence systems, the model can contribute to boosting human performance and accelerating human decision making [25].

We also presented the additional information of ε accuracies allowing one- or two-stage errors. We believe that providing sub-categories for each of the CS1~CS6 classes will help a lot of orthodontists in making a more accurate medical decision. For example, each class may have three sub-categories: low, middle, and high. One of our future areas of research is to extend the classes into sub-categories in order to consider the nature of the regression problem and to develop a suitable deep learning model.

The conventional methods rely on some manual operations in a series of processing steps such as inputting the specific landmarks. The manual operation is prone to lead to a subjective medical decision. It also hurts the reproducibility of the same performance on the same patient group. In most situations, the direct decision of the bone age is more accurate or easier than inputting extra landmarks. Since our method is fully automatic, it is meaningful to install the method in some commercial orthodontic systems. The obstacle to performance improvement in our study was the error of ε(2) or ε(3). Errors above ε(2) were observed to be 9% for model_1, 8% for model_2, and 6% for model_3, respectively. Although it does not occupy a high proportion in the overall error rate, it is an important evaluation criterion in terms of prediction accuracy. The ε(2) and ε(3) errors are mostly incorrectly evaluated as CS3 or CS4. In particular, the error of epsilon (1) was also observed to be the highest in CS3. The CS3 stage includes the peak period of puberty, and it has been reported that the prediction accuracy is low even in previous studies. In future research, it is necessary to reduce the ε(2) error and increase the prediction accuracy in the CS3 step.

A critical limitation of conventional studies and our study is the lack of state-of-the-art comparison of classification accuracy. This comes from the fact that no public datasets are available. Therefore, direct comparison of accuracy between studies is not allowed. On the other hand, the cephalometric landmark detection problem has a de facto standard dataset named ISBI2015 [26]. The ISBI2015 dataset has served for the Cephalometric X-ray Image Analysis Challenge held by the IEEE International Symposium on Biomedical Imaging and for comparing the state-of-the-art performance of the models proposed by academic papers [27]. One of the most important future works in CVM is to construct a publicly available dataset.

This study had several clinical limitations. Since this study used the panoramic image of a single medical institution, its generality is limited. In the future, it will be possible to improve the generalization performance of the model through objective evaluation using external validation data or a benchmark dataset. Moreover, it is not known whether the performance of the model is clinically acceptable, as no comparative studies of human doctors and models have been conducted. It is necessary to conduct comparative studies with clinicians through follow-up studies.

The future scope for research is as follows. Recently, 3D cephalometric computed tomography (CT) has become popular. We will enhance the performance by extending our methods to processing 3D cephalometric CT. A recent study conducted by the AO Spine Cervical Classification Validation Group [28] agreed to use MRI as a screening tool despite regional and empirical differences in surgeon preference for the treatment of cervical facet joint injuries. In order to secure the reliability of the estimation of cervical vertebrae maturity, it is better to use MRI or CBCT 3D data rather than 2D lateral cephalograms. Occasionally, congenital absence of the cervical spine pedicle has been reported [29,30]. If such a benign malformation is observed, there is a possibility of a diagnosis error because it could only be confirmed through three-dimensional CT reconstruction images. Another future study is to use automatic landmark detection techniques. Since accurate landmark detection algorithms are available, it is possible to combine landmark detection and bone age classification. We expect that this combination will significantly improve the bone age estimation accuracy. Finally, the explanatory power of the model will be visualized using the explanation of artificial intelligence such as a class activation map. Although it is important to classify the maturity in the cervical spine image, studies on a model that can explain the classification performance appropriately for clinical use will also be needed.

## 5. Conclusions

Most of the papers that evaluated bone age estimation using machine learning selected the hand, wrist, knee, or clavicle as the ROI [31], but in this study, deep learning was applied using cervical vertebrae for age estimation. This study described a dataset with 600 images and proposed three deep learning models that work automatically. Owing to the nature of gradual growth, we devised the ε(x) index, allowing one- or two-stage errors, and presented the accuracies of three classification models. Achieving more than 93% accuracy for ε(1), we demonstrated the practicability of the proposed approaches.

## Figures and Tables

**Table 1:** ε(x) accuracies of the three models.

ε(x)	Model		
	Model_1	Model_2	Model_3
ε(0)	0.5333	0.5916	0.6250
ε(1)	0.9250	0.9667	0.9333
ε(2)	0.9916	0.9833	0.9833

## Data Availability

The data sets used and/or analyzed during the current study are available from the corresponding author on reasonable request.

## References

[1] Martin D.D., Wit J.M., Hochberg Z., Sävendahl L., van Rijn R.R., Fricke O., Cameron N., Caliebe J., Hertel T., Kiepe D. (2011). The use of bone age in clinical practice—Part 1. Horm. Res. Paediatr..

[2] Greulich W.W., Pyle S.I. (1959). Radiographic Atlas of Skeletal Development of the Hand and Wrist.

[3] Tanner J.M., Whitehouse R.H., Cameron N., Marshall W.A., Healy M.J.R., Goldstein H. (2001). Assessment of Skeletal Maturity and Prediction of Adult Height (TW2 Method).

[4] McNamara J.A., Franchi L. (2018). The cervical vertebral maturation method: A user’s guide. Angle Orthod..

[5] Gabriel D.B., Southard K.A., Qian F., Marshall S.D., Franciscus R.G., Southard T.E. (2009). Cervical vertebrae maturation method: Poor reproducibility. Am. J. Orthod. Dentofacial. Orthop..

[6] Nestman T.S., Marshall S.D., Qian F., Holton N., Franciscus R.G., Southard T.E. (2011). Cervical vertebrae maturation method morphologic criteria: Poor reproducibility. Am. J. Orthod. Dentofac. Orthop..

[7] Spampinato C., Palazzo S., Giordano D., Aldinucci M., Leonardi R. (2017). Deep learning for automated skeletal bone age assessment in X-ray images. Med. Image Anal..

[8] Lee H., Tajmir S., Lee J., Zissen M., Yeshiwas B.A., Alkasab T.K., Choy G., Do S. (2017). Fully automated deep learning system for bone age assessment. J. Digit. Imaging.

[9] Kim J.R., Shim W.H., Yoon H.M., Hong S.H., Lee J.S., Cho Y.A., Kim S. (2017). Computerized bone age estimation using deep learning based program: Evaluation of the accuracy and efficiency. Am. J. Roentgenol..

[10] Gray S., Bennani H., Kieser J.A., Farella M. (2016). Morphometric analysis of cervical vertebrae in relation to mandibular growth. Am. J. Orthod. Dentofac. Orthop..

[11] Baptista R.S., Quaglio C.L., Mourad L.M.E.H., Hummel A.D., Caetano C.A.C., Ortolani C.L.F., Pisa I.T. (2012). A semi-automated method for bone age assessment using cervical vertebral maturation. Angle Orthod..

[12] Džemidžić V., Sokic E., Tiro A., Nakaš E. (2015). Computer based assessment of cervical vertebral maturation stages using digital lateral cephalograms. Acta Inform. Med..

[13] Moraes D.R., Casati J.P., Rodrigues E.L. Analysis of polynomial behavior of the C3 cervical concavity to bone age estimation using artificial neural networks. Proceedings of the 2013 ISSNIP Biosignals Biorobotics Conference: Biosignals and Robotics for Better and Safer Living (BRC).

[14] Kök H., Acilar A.M., İzgi M.S. (2019). Usage and comparison of artificial intelligence algorithms for determination of growth and development by cervical vertebrae stages in orthodontics. Prog. Orthod..

[15] Amasya H., Yildirim D., Aydogan T., Kemaloglu N., Orhan K. (2020). Cervical vertebral maturation assessment on lateral cephalometric radiographs using artificial intelligence: Comparison of machine learning classifier models. Dentomaxillofac. Radiol..

[16] Makaremi M., Lacaule C., Mohammad-Djafari A. (2019). Deep learning and artificial intelligence for the determination of the cervical vertebra maturation degree from lateral radiography. Entropy.

[17] Seo H., Hwang J., Jeong T., Shin J. (2021). Comparison of Deep Learning Models for Cervical Vertebral Maturation Stage Classification on Lateral Cephalometric Radiographs. J. Clin. Med..

[18] Baccetti T., Franchi L., McNamara J.A. (2002). The cervical vertebral maturation (CVM) method for the assessment of optimal treatment timing in dentofacial orthopedics. Angle Orthod..

[19] Lin T.Y., Goyal P., Girshick R., He K., Dollar P. (2018). Focal loss for dense object detection. IEEE Trans. Pattern Anal. Mach. Intell..

[20] Taghanaki S.A., Zheng Y., Zhou S.K., Georgescu B., Sharma P., Xu D., Comaniciu D., Hamarneh G. (2019). Combo loss: Handling input and output imbalance in multi-organ segmentation. Comput. Med. Imaging Graph..

[21] Deng J., Dong W., Socher R., Li L.-J., Li K., Fei-Fei L. ImageNet: A large-scale hierarchical image database. Proceedings of the 2009 IEEE Conference Comput Vision Pattern Recognit.

[22] Ronneberger O., Fischer P., Brox T. U-Net: Convolutional Networks for Biomedical Image Segmentation. Proceedings of the Medical Image Computing and Computer-Assisted Intervention (MICCAI).

[23] Oktay O., Schlemper J., Folgoc L.L., Lee M.J., Heinrich M., Misawa K., Mori K., McDonagh S.G., Hammerla N., Kainz B. (2018). Attention U-net: Learning where to look for the pancreas. arXiv.

[24] Schoretsaniti L., Mitsea A., Karayianni K., Sifakakis L. (2021). Cervical Vertebral Maturation Method: Reproducibility and Efficiency of Chronological Age Estimation. Appl. Sci..

[25] Topol E.J. (2019). High-performance medicine: The convergence of human and artificial intelligence. Nat. Med..

[26] Wang C.W., Huang C.T., Lee J.H., Li C.-H., Chang S.-W., Siao M.-J., Lai T.-M., Ibragimov B., Vrtovec T., Ronneberger O. (2016). A benchmark for comparison of dental radiography analysis algorithms. Med. Image Anal..

[27] Oh K., Oh I.S., Le T.V., Lee D.-W. (2021). Deep Anatomical Context Feature Learning for Cephalometric Landmark Detection. IEEE J. Biomed. Health Inform..

[28] Canseco J.A., Schroeder G.D., Patel P.D., Grasso G., Chang M., Kandziora F., Vialle E.N., Oner F.C., Schnake K.J., Dvorak M.F. (2021). Regional and experiential differences in surgeon preference for the treatment of cervical facet injuries: A case study survey with the AO Spine Cervical Classification Validation Group. Eur. Spine J..

[29] Perrini P., Montemurro N. (2016). Congenital absence of a cervical spine pedicle. Neurol. India.

[30] Tiyaworabun S.T., Beeko D., Bock W.J. (1982). Congenital absence of a pedicle in the cervical spine. Acta Neurochir..

[31] Dallora A.L., Anderberg P., Kvist O., Mendes E., Ruiz S.D., Berglund J.S. (2019). Bone age assessment with various machine learning techniques: A systematic literature review and meta-analysis. PLoS ONE.

